# The use of global positional satellite location in dementia: a feasibility study for a randomised controlled trial

**DOI:** 10.1186/1471-244X-14-160

**Published:** 2014-05-30

**Authors:** Heather Milne, Marjon van der Pol, Lucy McCloughan, Janet Hanley, Gillian Mead, John Starr, Aziz Sheikh, Brian McKinstry

**Affiliations:** 1 E-Health Group, Centre for Population Health Sciences, The University of Edinburgh, Telescot, Room 216b, Doorway 3, Medical School Teviot Place, Edinburgh EH8 9AG, UK; 2 Health Economics Research Unit, The University of Aberdeen, Aberdeen, UK; 3 The Edinburgh Health Services Research Unit, Edinburgh, UK; 4 Department of Nursing, Edinburgh Napier University, Edinburgh, UK; 5 Department of Geriatric Medicine, The University of Edinburgh, Edinburgh, UK; 6 Division of General Internal Medicine and Primary Care, Brigham and Women’s Hospital, Brigham, USA; 7 Department of Medicine, Harvard Medical School, Boston, USA

**Keywords:** Dementia, Feasibility study, Global positional satellite location, Wandering

## Abstract

**Background:**

Getting lost outside is stressful for people with dementia and their caregivers and a leading cause of long-term institutionalisation. Although Global Positional Satellite (GPS) location has been promoted to facilitate safe walking, reduce caregivers’ anxiety and enable people with dementia to remain at home, there is little high quality evidence about its acceptability, effectiveness or cost-effectiveness. This observational study explored the feasibility of recruiting and retaining participants, and the acceptability of outcome measures, to inform decisions about the feasibility of a randomised controlled trial (RCT).

**Methods:**

People with dementia who had been provided with GPS devices by local social-care services and their caregivers were invited to participate in this study. We undertook interviews with people with dementia, caregivers and professionals to explore the perceived utility and challenges of GPS location, and assessed quality of life (QoL) and mental health. We piloted three methods of calculating resource use: caregiver diary; bi-monthly telephone questionnaires; and interrogation of health and social care records. We asked caregivers to estimate the time spent searching if participants became lost before and whilst using GPS.

**Results:**

Twenty people were offered GPS locations services by social-care services during the 8-month recruitment period. Of these, 14 agreed to be referred to the research team, 12 of these participated and provided data. Eight people with dementia and 12 caregivers were interviewed. Most participants and professionals were very positive about using GPS. Only one person completed a diary. Resource use, anxiety and depression and QoL questionnaires were considered difficult and were therefore declined by some on follow-up. Social care records were time consuming to search and contained many omissions. Caregivers estimated that GPS reduced searching time although the accuracy of this was not objectively verified.

**Conclusions:**

Our data suggest that a RCT will face challenges not least that widespread enthusiasm for GPS among social-care staff may challenge recruitment and its ready availability may risk contamination of controls. Potential primary outcomes of a RCT should not rely on caregivers’ recall or questionnaire completion. Time spent searching (if this could be accurately captured) and days until long-term admission are potentially suitable outcomes.

## Background

Approximately 24 million people worldwide are known to have dementia [1] and many more are undiagnosed [2]. As the population ages, the prevalence is expected to increase rapidly. Globally, the number of people living with Alzheimer’s disease (AD), the commonest cause of dementia will rise to 106 million by 2050 [3]. Most people with dementia wish to remain at home, and health and social care services encourage this, particularly as long-term care is an increasingly scarce [4] and expensive [5] resource. A leading reason why people with dementia require long-term care is because of “wandering” i.e. leaving home without informing a caregiver, or alternatively getting lost while out [6]. About 40% of people with dementia get lost outside their home at least once [6], and just under 10% get lost on multiple occasions [7]. Wandering outside the home puts them at risk of exploitation and accidental injury [8], family members and other caregivers naturally become anxious spending long periods searching for them [6] and the police frequently need to be involved [7]. Often, however, the person does not walk far from home, may be in familiar territory, can find their way home and be at relatively low risk. These insights have led to calls to modify the current policy of restricting external access through locked doors [9]. Although wandering and getting lost are a cause of both patient and caregiver stress [8], this must be balanced against the potential benefits in terms of physical exercise, social contact, informal supervision by neighbours and local shopkeepers and the perception of autonomy [9,10] afforded by what some social care organisations have termed “safe walking” [11,12]. Fear of getting lost is a contributor to the marked reduction in out-of-home mobility [13] and restricted life space [14] associated with cognitive impairment [15]).

One possible intervention to support safe walking is the use of electronic location devices. The *Global Positioning System* (*GPS*) navigation is widely used, particularly in cars, and it is now also a common component of smartphones. In theory these devices enable the exact co-ordinates (down to a few metres) of a person who has a GPS receiving device to be detected and forwarded by GPS mobile phone signal to a central server, which can then can be superimposed onto an electronic map. Safe areas and times can be set up which allow the person to move appropriately through familiar areas (e.g. a bus route or a walk to the local shop), but will set off an alert if this geo-temporal limit (or “geo-fence”) is breached. Additional services including adding an operator to phone the person and using GPS to guide them home have also been described [16]. However, if the person is out of view of the satellites, (e.g. in cities with high buildings) the location system is less effective, nor will it work an area with poor mobile phone coverage (for example some rural areas).

Health and social-care professionals, have however expressed some reservations about the impact on civil liberties that these devices may have [17], however observational studies suggest that they are popular with family caregivers [18], and that civil liberty issues do not seem to be a particular problem particularly with older carers [19].

Others have suggested that the numbers of people likely to benefit and take up such a service is small (around 25% of wandering people), partly because for some people no degree of unsupervised walking outside is safe, and that the use of such devices may lead to an increased risk of accidents [20].

Commercial organisations in several countries now offer GPS location services. However, our literature searches identified no systematic evaluation of GPS in the context of dementia. It is unknown what impact the use of such devices has on important outcomes such as the time taken by caregivers to find “lost” people with dementia, caregiver stress and anxiety, accidents, delay of admission to long-term care and service resource use and if the cost of providing such services is likely to be recouped through resource savings down the line.

These questions need to be answered through a randomised controlled trial (RCT). However, before this could be carried out, a feasibility study is needed to determine the:

•likely available number of eligible patients, caregivers or other appropriate participants and the willingness of participants to be recruited and of social care staff to identify and recruit participants

•perceptions of participants as to what constitutes problematic wandering behaviour/getting lost, the perceived utility of GPS location and which types of people might benefit most from the intervention

•rates of retention, compliance, and completion of study questionnaires

•availability, usefulness and limitations of routinely acquired health service and social care data to assess outcomes

•standard deviation of outcome measures to inform estimates of sample size for a future trial

•time needed to collect and analyse data.

## Methods

This was an observational study using mixed methods, combining data from qualitative interviews and focus groups with questionnaire and resource use data.

### Ethical issues

Ethical approval was obtained from Scotland A National Health Service (NHS) research ethics committee (11/AL/0292). People with dementia consented for themselves if they had capacity (as assessed by a psychiatrist from the Scottish Dementia Clinical Research Network), otherwise consent was sought from the caregiver.

### Participants

#### Caregivers and people with dementia

Participants were recruited by social care collaborators in Edinburgh, East Lothian and Fife social services. These regions had recently started routinely providing GPS to suitable people with dementia.

#### Inclusion and exclusion criteria

Inclusion criteria: These were decided upon by the local social care teams. We considered all people with dementia (of any cause) who met the local criteria for the GPS service and were willing to use it. GPS was provided to them on the basis that they had a history of “wandering”, were deemed suitable for current best practice (door alarm, linked to call centre), had road safety skills and had a caregiver/relative who could charge the battery daily, ensure the person with dementia carried it and were able to search for them. We included patients in the study with all levels of dementia severity providing they met the above criteria. If, social services staff deemed someone suitable, and offered a GPS, they then asked permission to pass the potential participants’ contact details to the research team. The researcher then provided potential participants with the details of the study and invited them to take part.

Exclusion criteria: People were excluded if they were terminally ill, or had another major psychiatric illness unrelated to dementia, or if the caregiver was unable to consent or had no telephone.

#### Professional stakeholders

We interviewed professional stakeholders (i.e. social workers, community psychiatric nurses, occupational therapists, police officers and call centre staff). Police officers were included due to their key role when a person with dementia is reported missing. These stakeholders were identified through social services and professional networks.

### GPS location systems

There was a range of possible devices available that could be carried in pockets and bags, worn as watches, or as a pendant. However, the technology in each of the devices was similar and monitoring while carried out by different agencies was also similar. The social care providers chose a device from a range of possible devices that they had previously piloted, and which they considered would suit the individual patient best. Participants and their family caregivers were given instruction on applying the tracking system by the social care partners. “Geo-fences” were established in agreement with the person with dementia and their caregivers. They varied from a very wide area for a man who was a keen walker and for another participant who had a defined bus route, to the immediate environment of the family home in one case where the family did not trust the system and used it as a back-up to locked door. If the person with dementia was reported lost or had breached their ‘geo-fence’, the monitoring agency could inform the caregiver of their last known whereabouts detected by GPS and continue to inform them as needed if the person was moving. Caregivers could also track users on their computer, tablet and mobile phone.

### Duration of observation

It was intended that the GPS location devices would be provided by social-care staff for as long as they were deemed useful. However, we aimed to evaluate the intervention over approximately six months to allow a sufficient number of wandering events (as identified by the caregiver) and the possibility of some admissions to hospital or care homes or both to occur and also to give an indication of the likely retention of these devices. A previous local audit suggested around 20% of those meeting the inclusion criteria would have at least one admission during this period and previous research suggested that the median length of time between first wandering and institutionalisation was eight months [21].

### Frequency of follow-up

Participants were recruited throughout the period of the research project. We aimed to follow-up participants at six months for the main assessment, and also to contact them on two occasions approximately two months apart by telephone, to obtain health and social care usage data (see below). However, we accepted that for people recruited late in the period of the research project that the observation period and planned number of iinterviews would be curtailed. For those who requested it we agreed that the final assessment could be carried out by telephone.

### Testing the acceptability of baseline and outcome measures

#### Baseline measures

Around the time of receiving the GPS or shortly afterwards, we trialled the following instruments with people with dementia and their caregivers; Modified Caregiver Strain Index (mCSI) [22], Hospital Anxiety and Depression Score (HADS) [23] for caregiver, Mini Mental State Examination (MMSE) [24] for patients, Barthel Score [25] for patients, the Index of Capability for older people for both caregiver and person with dementia (ICECAP-O)[26], the Carer Experience Scale (CES) [27]. We also asked caregivers about the perceived frequency of wandering/lost episodes, estimated time spent searching and number of days spent in residential care in the previous six months.

#### Outcome measures at end of study

We repeated the baseline measures (apart from MMSE and Barthel) and also collected: adverse events e.g. accidents, injuries and falls reported by caregivers; number of episodes of wandering or getting lost documented by caregiver during the study period and detected by GPS; estimated number of hours searching for participants during the study period, and for one type of device, the number of triggers of geo-fence breaches from GPS records; number of participants admitted to, time to admission and number of days spent in nursing or residential homes or NHS long-term care facilities during the period of the intervention; number of attendances by family practitioner, out-of hours (OOH) calls, emergency room, outpatients and hospital admissions, number of recorded calls to social services.

#### Resource use and quality of life measures for use in economic evaluation

Wandering or getting lost represents only one of the reasons why people with dementia, many of whom have multimorbidity, may engage with health or social services. However given the potential for injury and the association with early admission to long term care we thought it useful to determine the feasibility of collecting data on health and social care use. Economic evaluation compared the costs and benefits of GPS with standard care. Costs included all health and social care resource use (i.e. general practitioners, hospital out-patient and in-patient episodes, emergency room and out-of-hours attendances, social care visits, admissions to care home). Resource use can be identified in a number of ways within a trial including: patient questionnaire, patient diary and patient records. These were piloted with caregivers within the feasibility study. Telephone questionnaires were scheduled every two months. In terms of patient records we attempted to obtain data from hospital, social care and general practice records. Response rates and where possible estimates of resource use were compared across the three methods i.e. telephone questionnaire, diary and patient records.

Economic evaluations tend to rely on generic quality of life measures such as the Euroqol-5D (EQ-5D). However, these are not appropriate in this context as they only capture health-related quality of life of the patient and not general quality of life and ignores any impact on the quality of life of the caregivers. We therefore explored the feasibility of alternative quality of life instruments which can capture important outcomes in patients and their caregivers namely the ICECAP-O capability index [26] and the Carer Experience Scale [27]. Population utility scores (on a scale from 0 to 1) are available making these instruments suitable for use in economic evaluation.

#### Time spent searching

Initially, we asked carers to estimate how frequently they felt the person with dementia was “wandering”, but they found this a difficult concept, because they wanted the person to continue walking independently. We therefore asked them to record in a diary how much time they spent searching for the person and how frequently they did this.

#### Method of assessing baseline and outcome measures

Participants were seen by the researcher at the participants’ homes to complete questionnaires or if not convenient for the caregiver the interview was conducted by telephone with the questionnaires having been previously posted to them to read. Electronic GPS records were analysed for breaches of the geo-fence to compare with caregiver recollection of “wandering” episodes, although we accepted that this might be an underestimate of concern about being lost as although people might not breach their geofence they would be late home. We recorded the views of caregivers and people with dementia on the obtrusiveness of the measuring process.

#### Study power and statistical analyses

This was a feasibility study designed to explore recruitment, the acceptability of the intervention and the research instruments, and provide some indication of the variance in outcomes to inform the power of a possible future trial. We aimed to recruit 20 people monitored for six months as this was considered to be sufficiently large enough to provide a representative sample of the kinds of people being offered this service and based in the audit above a realistic probability of episodes of getting lost and admissions to long-term care occurring. Simple descriptive statistics were used for feasibility and acceptability measures, and for variability of outcomes of interest including hospital and social care use, in order to inform the design of a larger study.

#### Qualitative evaluation

People with dementia and caregivers: We conducted semi-structured interviews with family-caregiver/participants with dementia at the time of receiving the GPS device or shortly afterwards to explore their hopes and concerns about the GPS and what safe walking meant to them. We planned to re-interview caregiver/participants between 4–6 months later to find out how the intervention had affected them, however some were recruited so late in the study period it was considered that an interview so soon after the first would be unduly burdensome. Family-caregivers and participants with dementia were given the choice of combined or separate interviews. We had intended a purposive, maximum variation sampling approach, but due to slow recruitment, we interviewed all available willing participants and caregivers in order to gain a full range of experience/perceptions and maximise the chances of data saturation.

Professionals: In order to determine the views of social care, police and healthcare professionals of the acceptability and utility of satellite location and conduct of a RCT we undertook a focus group with additional one-to-one interviews for those who could not participate in the focus group. Focus group dynamics may reveal aspects of a topic or reveal information that may not have been anticipated by the researcher or have emerged from individual interviews. The focus group and interviews were conducted towards the end of the data collection period. The interview guides were based both on the themes identified from the literature and informed by initial analysis of data from qualitative interviews with the GPS users.

Data handling and analysis: Transcribed interviews were entered into Nvivo 9 and subjected to iterative analysis employing constant comparison across the various interviews and focus groups to ensure that the thematic analysis represented all perspectives [28].

## Results

### Recruitment and retention of people with dementia and their caregivers

In planning the study, social care services had estimated at least 50 people would be offered the GPS device during the recruitment period of the study of which we had planned to recruit 20. However, over the eight month recruitment period, only 20 devices were offered to clients by social care services in Fife, Edinburgh and East Lothian (population 1.5m). This was because fewer people than expected were referred for, or requested GPS and met social services assessment criteria. Of these fourteen were referred to the study and 12 (86%) caregiver/client dyads were recruited. We were unable to discover why six people assessed by social services did not wish to be referred to the study. Reasons for the two non-participating referrals were: “withdrawal of service due to absence of caregiver willing to apply the device” and caregiver concerns that use of the term “dementia” in interviews might upset the client. Several people were referred quite late into the recruitment period and so were in the study for less than six months; the minimum period of follow-up was six weeks. This meant that the full number of planned interviews did not take place as there was insufficient time to do so. Four people received two telephone follow-ups, six received one and two had no interim telephone follow-ups. Three caregivers asked for the final interview to be carried out by telephone as they felt it would be easier for them given the deterioration in the person with dementia who they were looking after.

### Recruitment into the qualitative evaluation

Twelve caregivers and eight people with dementia had first interviews and eight caregivers and four people with dementia were available for a follow up interview. People with dementia did not take part because of cognitive decline, physical ill health and, in one case, refusal to use the GPS. One caregiver declined a second interview, another could not be contacted and two were recruited late in the study with little time for the effects of the intervention to be established and so were only interviewed once. The time spent in the study and types of devices allocated to participants and their characteristics are reported in Table 1. The characteristics of the people with dementia and their caregivers recruited to take part in interviews are reported in Table 2.

**Table 1:** The types of devices, support and configuration of geofence of the participating people with dementia

**ID**	**Time in study**	**Type of device**	**Had geofence**	**Area description**	**Furthest distance from home before alert**	**Reasons given for this configuration**
	**Months**		**Yes/no**			
**P01**	6	VEGA support by BIELD	y	To local shops	500m	- Area usually walked in recently
						- No major roads
						- Big enough to choose different routes
**P02**	7	Buddi	no			As soon as she started walking down the road her husband would go after her
**P03**	7	Buddi	y	Did not know what had been set at baseline		
**P04**	5	VEGA support by BIELD	y	Centre of Edinburgh and main shopping centres	5 km	- Area usually walked in recently
						- Familiar area/comfort zone – known for many years
**P05**			na			
**P06**	4	VEGA support by BIELD	n			- Legitimate outings would set it off
**P07**	4	ROMAD from TUNSTALL support by GEONOVO	y	Along main roads to the neighbouring towns	7km	- Area usually walks
**P08**	4	SKYGUARD x 2	n			- Too restrictive
**P09**	4	BUDDI	n			- Legitimate outings would set it off
**P10**	4	VEGA support by BIELD	y	Edinburgh	8km reduced to 5km	- Boundary wasn’t a key concern as he covers a wide area
**P11**	3	SKYGUARD	n			- Legitimate outings concern that may not be able to contact call centre to turn off boundary
						- Too restrictive
**P12**	1	VEGA support by BIELD	y	Originally limited to their road then extended to whole of Edinburgh	? 8km	- Area usually walks in (to supermarket)
						- Familiar area – because likes to visit other parts of Edinburgh where grew up

**Table 2:** Characteristics of caregivers and people with dementia who participated in interviews

	**Person with dementia**					**Caregiver(s)**	**Research involvement**
**ID**^**a**^	**Age**	**Dementia diagnosis**	**Baseline MMSE**	**Baseline BARTHEL**	**Consent**	**Relationship to dementia sufferer**	**Time in study**
C01P01	70-74	Vascular	18	95	Consented	Husband	6 months
C02P02	60-64	Alzheimer’s	15	70	Consented	Husband	7 months
C03P03	70-74	Alzheimer’s	15	55	Consented	Wife	7 months
C04P04	75-79	Unknown	21	65	Consented	Daughter and her husband	5 months
C05	Unknown				-	Wife	Baseline only^a^
C06	55-59	Vascular	-	55	-	Sister	4 months
C07P07	70-74	Alzheimer’s	18	85	Proxy	Wife	4 months
C08P08	90-94	Lewy Body	26	100	Consented	Son and his wife	2 months
C09P09	70-74	Unknown	14	80	Proxy	3 sons	4 months^c^
C10P10	60-64	Alzheimer’s	Declined	Declined	Consented	Partner	4 months
C11P11	75-79	Vascular	25	90	Consented	Daughter	3 months^b^
C12	80-84	Alzheimer’s	-	-	-	Wife	1 month^b^

C = Caregiver; P = Person with dementia (if participated); ^a^Subsequently refused to use GPS. ^b^Baseline interviews only conducted.

### Recruitment of professionals to the focus group and interviews

Table 3 outlines the characteristics of the professionals recruited. One focus group and four face-to-face interviews for those who could not attend the group were undertaken.

**Table 3:** Professional stakeholders recruited

	**Number**
**Social service staff**	5
**Occupational therapists**	4
**Community Psychiatric Nurse**	1
**Call Centre Operators**	2
**Police Officers**	3
**Total**	15

### Results from the qualitative interviews

Perceptions of the concepts of wandering and safer walking:

In qualitative interviews caregivers and people with dementia defined “wandering” as becoming lost/distressed and/or the caregiver being concerned about their whereabouts when they were not at the time or place where they were expected. Particular concerns included distress for the person with dementia, accidents and exploitation.

“You do hear such horrific stories of old people, going missing and never being found again.” (Care giver (C)4)

Despite these concerns, to many, walking was important and should be continued if possible.

“There’s a risk but it’s worth taking” (Caregive (C) 1)

No, no, no, no, no. They’d never make you stop walking, I think that would be… (laughter) Wouldn’t go down well…you like walking, (C 10)

Hence, the aim of the GPS for social services and most service users was “safe walking”. This meant enabling the person to continue to walk independently by managing the perceived risks outlined above. “Safe areas” were those where the person with dementia was thought unlikely to become lost or risk exposed. Six of the 12 caregivers set a GPS “geo-fence” so they were alerted if there was a departure from the safe area which varied in size from a few streets to the whole city.

### Perceived utility and acceptability of using GPS location technology

Caregivers aimed to use the GPS for three overlapping purposes, which in turn influenced the outcomes which were considered important and acceptable to them. One person with dementia declined to use the GPS after it was provided by social services because he felt it was an unnecessary intrusion. Seven aimed to support independent “safe walking”, but of these only four successfully used the GPS for this purpose during the study because, for example, the person with dementia had become too nervous to walk alone. Two caregivers used them to monitor unscheduled walking; two caregivers felt despite the technology it was not safe to allow the person with dementia to go outside alone.

*“I don’t want to take his freedom away, [or] say ‘You can’t do that, you can’t do that cause it’s not safe for you.’ But now he’s able to do that because the, the tracker’s there you know.”*(C6)

Walking was seen as important by many caregivers and they felt reassured by having the GPS locator.

*“It’s really a big weight off my mind. To think that I can go on [the computer] when he’s late in coming in and see where he is… Because before I’ve gone out in the car and looked for him… it’s a big difference.” (*C7)

Three participants at baseline also expressed concern about being reminded of their condition and feeling watched, but when interviewed at follow up one of these said she had come to feel reassured by the GPS.

*“I did nae like it at first I thought, ‘This is absolutely terrible!’ …I did nae wanna know that I was gettin’ old and stupid.”* (P4)

Caregivers and professionals were generally enthusiastic about the benefits of the GPS for those in the less advanced stages of dementia, and highlighted positive outcomes such as “peace of mind” and the facilitation of allowing more freedom.

“…there’s certainly, … a lot of benefit to the GPS device as an enabler, to enable somebody to continue doing the things that they enjoy doing… They continue being out and about, there’s the health benefits associated with that, but also there’s, the carer’s stress is the other side of things and we’ve found, is generally, erm considerably reduced”. (Social Worker (SW)3)

Some caregivers and social care workers stated that without the GPS they would have had to limit the person’s freedom.

“It was either, she would have to be in a locked ward which would have been hugely distressing or, it was GPS.” (SW1)

The exceptions were two caregivers who used the GPS as a backup to locked doors. One did not trust the device and the other felt the person’s dementia was too advanced to safely walk outside. Some professionals were concerned that the use of GPS might create a false sense of security.

“…just because we know where someone is, do we know what risk they’re in?” (Police Officer1)

[people might think] “*Now we don’t need to use a carer because we’ve got a GPS and they can just go themselves.” (SW1)*

Some people with dementia and caregivers had lost confidence in walking independently and felt the device had come too late.

*“She’ll just be endangering herself if she had more freedom, other cases yeah I can see it working [for more independence], when they’re, when they’re not at the stage of dementia that she’s at.”* (C9)

There were complaints that the equipment was bulky and difficult to charge. The location was sometimes imprecise, failed to work everywhere and false alerts also occurred. Also there was a time delay which meant that the last recorded location wasn’t always where the person was if they were moving.

“[I got a phone call to say] ‘he’s out of boundary.’ An’ I said ‘Well, I find that quite strange because, I’ve only been out the house five minutes. An’ he would nae have time to go anywhere.’ … [so] I’ll go right back to the house and check that he’s, still there.’ ‘Oh,’ she says ‘If you would because…’ she says ‘I’ve got him down as being in the ocean.” (C7)

*“I have no faith in it whatsoever… [P03] wore it when he went to … day-care and Buddi [the GPS device] didn’t … tell me that he was on the move… it doesn’t work in the village…”* (C03)

Professionals were concerned that the GPS location system wouldn’t take into account weather conditions, nor identify that a person was insufficiently clad. In addition the system relied on a robust response service which could not always be provided by an elderly caregiver and reliable systems including police back-up must be part of the planning of any service.

A fuller description of the qualitative interviews and focus groups will be published in due course.

### Feasibility of proposed outcome measures

#### Caregiver stress and quality of life measures (mCSI,HADS, ICECAP-O and CES)

Ten people completed mCSI at baseline and five at follow up. Their scores indicated moderate caregiver strain at both baseline and follow up (Table 4).

**Table 4:** mCSI scores

	**mCSI baseline**	**mCSI follow up**
	**n = 10**	**n = 5**
**MEAN**	15.8	16.2
**SD**	4.6	4.6

The same 10 people completed HADs at baseline and five at follow-up (Table 5). Most caregivers who completed the HADs scored above the thresholds associated with clinically significant mood disorder at baseline and follow up. While it is feasible to conduct the mCSI and HADs in caregivers the refusal/drop-out rate was high.

**Table 5:** HADS scores

	**Anxiety baseline**	**Anxiety follow up**	**Depression baseline**	**Depression follow up**
	**n = 10**	**n = 5**	**n = 10**	**n = 5**
**MEAN**	12.2	13.4	8.9	10.6
**SD**	6.8	4.7	6.6	4.5

The CES was acceptable to caregivers, but several caregivers criticised the ICECAP-O for having unclear Likert scales and questions that did not seem relevant.

“I think that one where it’s looking, the one that’s looking at degrees, it’s quite a difficult thing to fill in that one…” (C8)

At baseline, nine people completed both the CES and ICECAP, but at follow-up only five people completed both measures (see Table 6).

**Table 6:** Caregiver ICECAP-O scores and caregiver CES scores

	**ICECAP-O**	**ICECAP-O**	**CES**	**CES**
	**Baseline**	**Follow up**	**Baseline**	**Follow up**
	**N = 8**	**N = 5**	**N = 9**	**N = 5**
**Mean**	0.62	0.58	0.61	0.58
**SD**	0.15	0.21	0.21	0.25

“You know, the stress you’re living under, you just don’t have the time to do it.” (C10)

Four people with mild dementia who were not suffering from visuo-perceptual difficulty attempted the ICECAP-O, but all struggled because it required them to remember several statements at once and recall instructions resulting in incorrect completion.

### Time spent searching

Caregivers were just too busy to keep diaries and so we asked them to estimate the time they spent searching. Estimates by caregivers of the approximate length of time spent searching for each episode where they feared the person with dementia was lost appeared to show a considerable reduction in estimated time spent searching when using the GPS (see Table 7). Data on the frequency with which people went outside their geo-fence were also feasible to extract from the GPS call-centre records. We carried this out for one type of device in three people (see Table 8). This showed a considerable variation from zero to 168 breaches during the observation period. The latter person who liked to walk everyday had started to decline cognitively quite markedly over the last two months he was in the study and increasingly frequently became lost requiring police intervention on one occasion when his GPS battery failed.

**Table 7:** Search times before and after GPS device

	**Before GPS**		**With the GPS**	
**ID**	**Typical time searching based on caregiver estimates from recall**	**Frequency searching**	**Typical time searching, based on caregiver estimates**	**Frequency of searching**
**1**	4 hrs	1 event	0	0
**2**	1 hour	2 or 3 times a week	10-30 mins	2 a month
**3**	2 hrs 30	1 event	0	0
**4**	6 hours	1-2 times a week	30 - 45 mins	1 or 2 a week
**5**			-	-
**6**	3 hours +	Walking at night	1 hour	1 a month
		Unclear how often		
**7**	20 mins	1 or 2 occasions/month searching plus walks taking longer	10-20 mins	2 a month
**8**	No searching but had been brought home a few times by others and described getting lost herself	1 or 2	No searching- used to monitor	
**9**	No missing/searching but was found at 4am around the back of the bus station	1 event	0	0
**10**	19 hours	Occasional 1 major missing event	45 minutes	At follow up, 2 or 3 times a day
**11**	20 minutes – and P has described being lost	Occasional	0	0
**12**	6hrs 30	2 main events	0	0
**MEAN**	4 hrs 44 (2 hrs 56 excl. 19 hr)		40 mins	

**Table 8:** Number of breaches of geo-fence, emergency button alerts and caregiver contacts

**Month**	**Nov-11**	**Dec-11**	**Jan-12**	**Feb-12**	**Mar-12**	**Apr-12**	**May-12**	**Jun-12**	**Jul-12**	**Total**
	**No. times out of geofence**									
**P1**	0	0	0	0	0	0	0	0	0	**0**
**P4**	0	1	1	6	2	1	3	3	1	**18**
**P10**				3	1	2	1	9	48	**64**

Caregivers reported that in addition to searching when the person with dementia went outside their geo-fence they searched when they did not arrive on time, suggesting these data may underestimate searching time. It is difficult to be sure how accurate caregivers’ recall of searching time was. Asking them to keep a written record was not successful. This may limit the usefulness of the length of time spent searching as a useful outcome measure in future research.

### Time to admission to long-term care and hospital

No-one was admitted to long-term care during the course of the study. However four people were admitted to hospital, one particularly prolonged admission (81 days) was for terminal care for cancer, one because she had some falls (on a background of myeloma), one with an infected wound and one unknown cause. One patient was admitted to respite care for seven days.

### Resource use and quality of life measures for use in economic evaluation

The use of patient diaries to estimate resource use was found not to be feasible with only one out of twelve diaries being returned. Caregivers indicated they were too busy to complete them. The response rate in telephone questionnaires was also relatively low (50%). Moreover, arranging to conduct the telephone interview at regular two month intervals was difficult. Only one caregiver completed the telephone questionnaire at a roughly two month intervals. Three caregivers completed the telephone questionnaire twice and two caregivers completed it once. The questionnaire took around 5–10 minutes to complete. Caregivers found questions about “wandering episodes” difficult to answer because they did not reflect the use of the GPS for “safer walking”. It was also found that more detailed questions should be included on the range of services available e.g. the use of specialist dementia nurses, police, befriender services, “Alzheimer”s buddies”.

Gaining access to GP patient records was feasible. All but one of 12 family practices asked to retrieve electronic data pertaining to general practice resource use did so. Data on hospital care were obtained for all patients. Whilst gaining access to social service records was feasible, abstracting the data was challenging. The records were mainly free-text so data had to be extracted manually; this took a mean 1.5 hours per client and required the services of a social care officer. It was also found that many types of interaction with clients such as social-worker and occupational therapist visits were not recorded. Only those activities which are purchased by social care appear to be accurately recorded.

### Comparison of carer recall of resource use with patients’ records

A comparison could only be done for hospital and GP records and robust individual level comparisons could not be made due to differences in samples and length of follow-up (mean was 5 months in telephone questionnaires and 5.5 in patient records). Table 9 shows that the average total costs are higher when using patient questionnaires, £7,714 per patient in case of patient questionnaires and £6,508 in case of patient records. However, while average costs were similar, individual costs varied considerably between the carer record and the hospital and GP record.

**Table 9:** NHS and social care resource use based on patient questionnaires and patient records

	**Unit cost £**	**Telephone questionnaire (N = 6)**		**Patient records (N = 9)**	
	**Unit cost**	**Quantity**	**Cost (£)**	**Quantity**	**Cost (£)**
AE	130	0.5	65.0	0.3	43.3
OPD	67	3.3	223.3	2.0	134.0
Inpatient days	384	17.2	6592.0	15.8	6058.7
Day case	498	0.2	83.0	0.2	110.7
GP surgery	36	3.0	108.0	1.3	48.0
GP Home visit	120	0.0	0.0	0.3	40.0
GP telephone	22	2.2	47.7	1.7	36.7
PN surgery	12	2.2	26.0	1.6	18.7
PN telephone	4	1.3	5.3	0.0	0.0
DN visit	27	0.8	22.5	0.7	18.0
DN telephone	7	0.2	1.2	0.0	0.0
Out of hours		0	0	0	0
**Total**			7174.0		6508.0

Table 8 shows the mean ICECAP-O and CES utility scores for caregivers. The mean scores were very similar for the two instruments.

## Discussion

This mixed methods study explored the feasibility of conducting a RCT on the impact of GPS location devices on caregivers’ quality of life and NHS and social care resources. Overall the study highlighted the many challenges facing such research in terms of recruitment, retention, burden on participants, and suitability of outcome measures particularly in relation to quality of life, but also in relation to the availability of resource use data.

Among the people we recruited, GPS systems to support “safe walking” were considered both useful and acceptable to caregivers and people with dementia. “Safe walking” meant supporting the person with dementia in independent walking while managing the risks associated with that and reassuring them and their caregivers. This is consistent with calls to rethink traditional definitions of “wandering” as a problematic behaviour to be limited or prevented [9,29-31]. These findings contribute to the small, but growing body of evidence on the acceptability of this technology to caregivers and people with dementia [18,32,33] by including the views of people at a range of stages of dementia and focusing on the provision of the GPS as a social care service.

A much smaller number of people than expected were considered suitable for the technology by social services, and, of those whom were given equipment to use, some did not use it to monitor or facilitate walking as either they or their caregivers had lost confidence in independent walking by the time they were given the equipment. This suggests that either a much larger geographical catchment area would be required to mount a substantive trial or that patients at an earlier stage of dementia should be included. This however, could reduce the effect size. In addition those recruited used a variety of different systems to suit their preferences. While this might have implications for potential heterogeneity of impact the systems basically all worked in the same way varying mainly in terms of format and to a lesser extent battery life. Our pragmatic study therefore replicated the likely diversity in devices that would be used in any future trial that recruited over several geographical areas.

The questionnaires (mCSI, HADS, CES) were largely acceptable except for the ICECAP-O for dementia sufferer and alternative QoL measures should be explored. The DEMQOL and DEMQOL-Proxy may be more appropriate [34,35]. However, there was considerable drop-out in the questionnaire response suggesting that any primary outcome measure in future research should not rely on data obtained from the person with dementia, and/or questionnaires should be designed and piloted to improve acceptability.

Caregivers were able to report the length of time they typically spent searching for the dementia sufferer when they were perceived as lost but sometimes only in broad terms rather than in actual hours of search. Our study suggested a reduction in searching time with GPS, but it was difficult to verify this. In the small group of people for whom geo-fence breaching data was available the patient-reported search burden broadly reflected the reported data breach frequency. However, there was a big variation in the amount of time spent searching which would suggest future substantive studies would have to be large to have sufficient power to detect a difference in this outcome. Although our small sample size does not allow for generalisable conclusions this is consistent with other research [36]. Time spent searching is a potential outcome for future research, but further work in a pilot trial would be needed to further explore better methods of recording this (for example by sending a text to a search service).

Diaries to measure resource use and time spent searching were not found to be feasible and although the telephone questionnaire was acceptable, conducting this on a bi-monthly basis was challenging in terms of caregivers availability. Extracting data from computerised secondary and GP healthcare records was feasible, but automatic extraction of data from social care records was not. Alternative ways of collecting these data need to be explored. In any case it is not clear to what extent wandering or getting lost contributes to overall expenditure and it would be difficult to extract this from the data.

No-one was admitted to long-term care during the course of the study despite suggestions from the literature [21] and audit with previous patients in SE Scotland and the literature that around 20% of the sample would expect to be admitted. This may be because the criteria for admission had become stricter, general home care support had become more effective or that there may have been an effect of using the GPS. Potentially, however, long-term institutionalisation remains a candidate for a primary outcome of a RCT. Social services locally indicated that the longest period of someone using the GPS device before entering long term care was a year so the data collection period of six months may have been too short or the sample too small to capture this. Time spent in respite care may also be a useful outcome.

Finally, the qualitative interviews revealed that social care staff were often convinced of the benefits of GPS and may therefore be unwilling to randomise people with dementia to a control intervention at this stage of dementia. For this reason, provision of devices at an earlier stage in the dementia trajectory, for example at memory clinics, where randomisation may be more acceptable should be considered. However, such devices are now commonly included in smart phones and the technology is relatively easy to purchase, which may lead to contamination of any control group who by dint of volunteering for a trial would be clearly interested in the technology and wish to use it regardless of randomisation. Alternative research designs such as an interrupted time series [37] may therefore be more appropriate, although the progressive nature of the illness presents a problem for this type of design. Participants would have to be observed without GPS for a period (which may not be acceptable to caregivers) and this observation period could not be too long as to allow the disease to progress potentially affecting results during the intervention period.

## Conclusions

GPS location has the potential to improve the lives of people with early dementia enabling them to keep active for longer. Conducting a RCT will be challenging in terms of finding social care staff willing to recruit, and randomise participants and furthermore finding participants/caregivers who are willing to participate as controls. Outcome measures which minimise burden to the participants and preferably do not rely on participant recall should be utilised, however, current social care records, will pose a challenge to accurate calculations of resource use. Delay to long-term admission or reduction in day care are possible primary outcomes as these are well recorded. Although imprecisely estimated, time spent searching, seemed to be greatly reduced by the technology; alternative more objective ways to accurately capture this should be sought. Future research should potentially focus on people at an earlier stage of dementia, where randomisation may be more acceptable, but the trade-off is that the effect size is likely to be diminished. The increasing penetration of GPS location technology into everyday use will pose a risk of contamination of the control group.

## Abbreviations

CES: Carer experience scale; DEMQOL: Dementia quality of life instrument; EQ-5D: Euroqol-5D; GPS: Global positional satellite; HADS: Hospital anxiety and depression score; ICECAP-O: Index of capability for older people; mCSI: Modified caregiver strain index; MMSE: Mini mental state examination; NHS: National health service; QoL: Quality of life; RCT: Randomised controlled trial.

## Competing interests

The authors declare that they have no competing interests.

## Authors’ contributions

HM conducted the interviews and analysed the data; MvdP advised on the health economic aspects of the study and helped analyse the data; LMcC was project manager for the study; JH advised on the qualitative aspects of the project and helped plan the research; GM helped plan the research and advised on methods; JS helped plan the research, facilitated the network involvement; AS helped plan the research and advised on methods; BM was chief investigator, planned the research and wrote the initial draft of the paper. All authors contributed to the writing of the paper. BM is guarantor. All authors read and approved the final manuscript.

## Authors’ information

Dr HM is a qualitative researcher and is currently now a medical student; Prof MvdP is professor of Health Economics at Aberdeen University; Dr LMcC is programme manager for the eHealth Unit in Edinburgh University; Dr JH is reader in nursing research at Edinburgh Napier University; Prof GM is professor of Stroke and Elderly Care Medicine in Edinburgh University; Prof JS is Honorary Professor of Health & Ageing at Edinburgh University, Prof AS is professor of primary care research at Edinburgh University and Harkness Fellow in Health Care Policy and Practice, Brigham and Women's Hospital/Harvard Medical School; Prof BM is professor of primary care eHealth at Edinburgh University.

## Pre-publication history

The pre-publication history for this paper can be accessed here:

http://www.biomedcentral.com/1471-244X/14/160/prepub
